# Management of a Severely Submerged Primary Molar: A Case Report

**DOI:** 10.1155/2013/796242

**Published:** 2013-04-22

**Authors:** Iman Parisay, Fatemeh Kebriaei, Bentolhoda Varkesh, Milad Soruri, Roya Ghafourifard

**Affiliations:** ^1^Pediatric Dentistry, Dental Material Research Center, School of Dentistry, Mashhad University of Medical Sciences, Mashhad, Iran; ^2^Department of Pediatric Dentistry, School of Dentistry, Shahid Sadoughi University of Medical Sciences, Yazd, Iran

## Abstract

Ankylosis is a condition frequently associated with primary molars, wherein the ankylosed primary teeth remain in a fixed position, while the adjacent teeth continue to erupt, moving occlusally. In this case report, a five-year-old boy, who had a retained and submerged left lower second primary molar, was presented. Luxation of ankylosed primary molar was considered as a treatment approach. After four months, the tooth erupted to the occlusal level, and there was evidence of further development of a permanent successor in radiographic evaluation. After one year, tooth mobility, bone formation, and development of a permanent successor were in good condition.

## 1. Introduction

*Dental infraocclusion* is defined as teeth below the occlusal plane. In the literature, the terms submergence and infraocclusion are often used to refer to an ankylosis [1, 2].

The frequency of ankylosed teeth has been reported to be between 1.3% and 38.5% [3]. The mandibular first primary molars are the most frequently affected teeth, followed by second mandibular and maxillary primary molars [3].

The exact cause of teeth ankylosis is still unknown, but several theories have been proposed [3, 4] such as familial pattern, traumatic injury to Hertwig's epithelial root sheath, deficiency in bone growth, a problem in local metabolism and inflammation, localized infection, and chemical or thermal irritations.

Ankylosis is classified as slight, moderate, or severe according to the place of the occlusal level of the infraoccluded tooth [5]. If the infraocclusion is less than 2 mm, it shows slight ankylosis, while moderate submergence shows the occlusal surface of the ankylosed tooth to the contact area. Severe ankylosis shows infraocclusion below the contact area of the adjacent teeth [5].

Diagnosing ankylosed teeth is not difficult and is usually based on clinical signs and radiographic findings. Clinically, ankylosed teeth have a sharp, solid sound on a percussion test in comparison to a cushion sound in normal teeth [2].

Obliteration of the periodontal ligament space is noted radiographically. The roots are less radiopaque, and as the ankylosis progresses, they are less distinguished from surrounding bone. Areas of fusion of cementum and bone, as well as periodontal ligament remnants that are fibrotic with very few cells, have been observed histologically. No mucopolysaccharidase activity, which is essential for the normal process of root resorption during eruption of permanent successor, is seen [6].

Ankylosis of deciduous molars has a negative impact on normal occlusal development and may cause problems such as

- significant tipping of adjacent teeth to the area of the submerged tooth, which may cause a reduction in arch length, especially when severe ankylosis of second primary molars occurs in early mixed dentition [5, 7];
- ectopic eruption or impaction of successor premolar;
- The increase in caries and periodontal disease susceptibility [3].

Hence, early diagnosis of infraoccluded primary molars is extremely important to prevent the previously mentioned problems.

There are several treatment procedures according to the age of the patient, the amount of tilting of adjacent teeth, and the condition of the permanent successor as follows:

- monitoring the ankylosed tooth [7];
- early extraction and space maintenance [5];
- restoration of occlusal height [8];
- luxation [3].

Here is a report of a case with ankylosis of the second primary molar in late primary dentition with a new treatment approach.

## 2. Case Report

A five-year-old boy was referred to the Pediatric Dentistry Department of Shahid Sadoughi University of Medical Sciences by his general dentist to assess his infraoccluded primary mandibular molar.

The patient had no relevant medical history of any systemic disease and his family reported no drug use.

**Figure 1:**
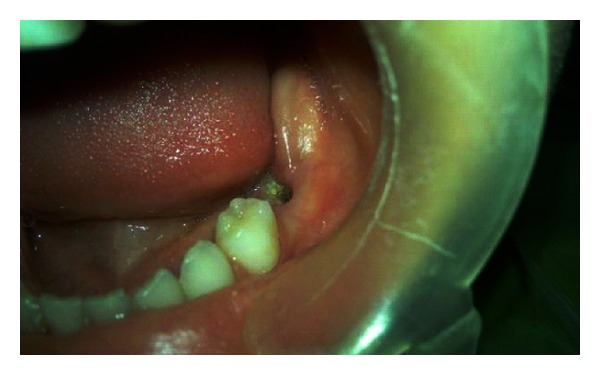
Intraoral view of the left second submerged primary mandibular molar.

Intraoral examination revealed multiple carious lesions in his maxillary anterior teeth and a severe infraoccluded left second primary mandibular tooth, the mesiolingual cusp of which was the only cusp that could be seen (Figure 1).

**Figure 2:**
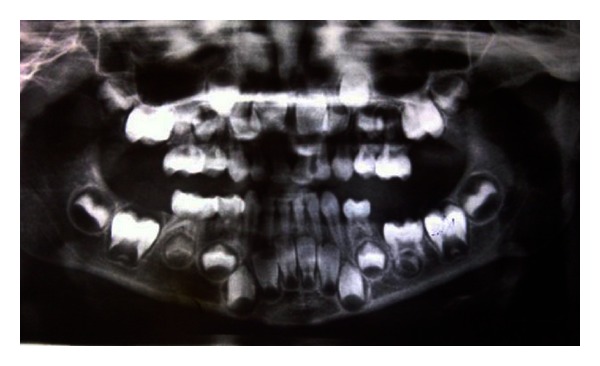
Radiographic view of the left second submerged primary mandibular molar.

A subsequent radiographic examination revealed an ankylosed second primary molar along with less development of a successor tooth bud in comparison with the antimer. The second premolar dental follicle was completely situated between the roots of the ankylosed primary molar (Figure 2). 

**Figure 3:**
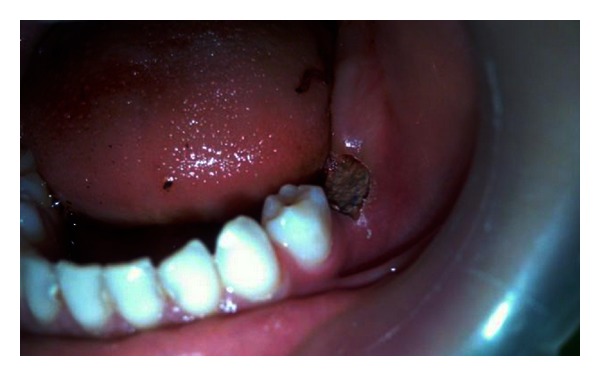
The surgical site was packed with reinforced zinc oxide eugenol.

Considering the abnormal development of the patient's permanent successor tooth, emergency intervention seemed logical. In order to prevent serious damage to dental follicle, surgical extraction was not considered; therefore, conservative intervention was a preferred treatment approach. The occlusal table of the submerged tooth was exposed by removing covering gingiva, and the surgical site was packed with reinforced zinc oxide eugenol (Zonalin, Kemdent, UK) to provide a path for the tooth to erupt (Figure 3). A carious lesion was detected in the central pit of the occlusal surface.

After one week, the ankylosed tooth was luxated mesially and distally by a surgical elevator, and because of no clinical or radiographic evidence of tooth eruption, after one month, the tooth was luxated for a second time.

**Figure 4:**
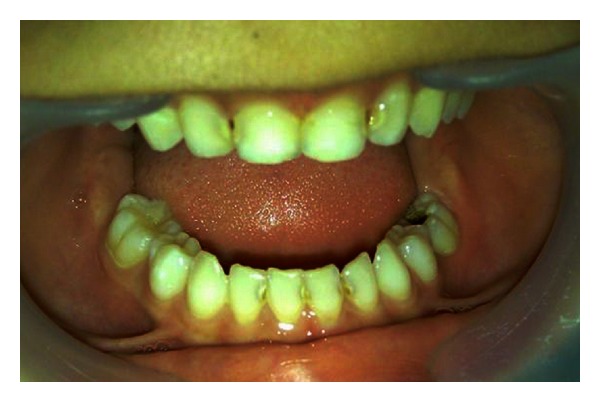
The tooth position four months after luxation.

After four months, the primary tooth erupted and reached the occlusal level of the primary dentition (Figure 4). There was evidence of further developments of a permanent successor in radiographic evaluation.

**Figure 5:**
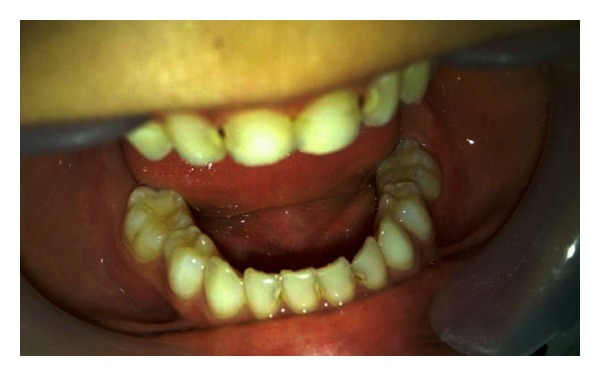
Restored tooth with resin composite.

Following the complete eruption of the primary tooth, the carious lesion was restored with posterior resin composite (P60, 3M ESPE, USA)  (Figure 5).

**Figure 6:**
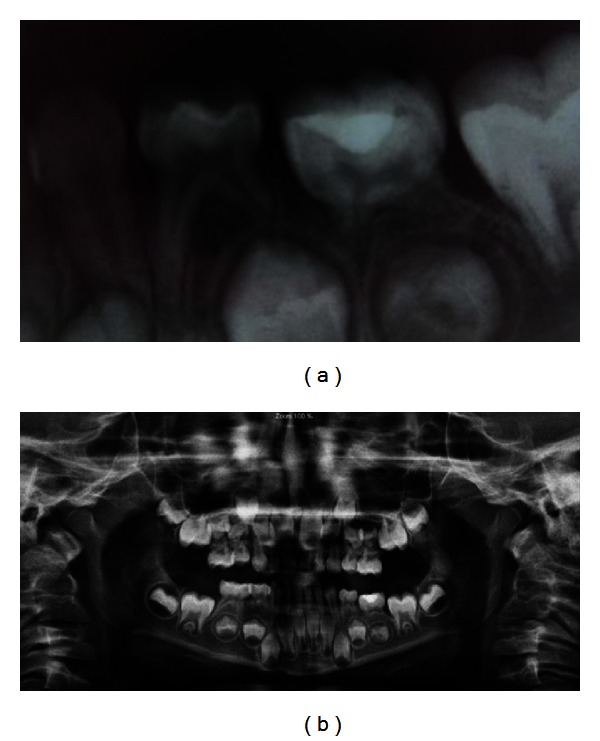
(a) Ten-month periapical view. (b) Ten-month panoramic view.

A favorable result was seen regarding tooth mobility, bone formation, and the development of a permanent successor in ten-month followup (Figure 6).

## 3. Discussion

In the management of an ankylosed tooth, early recognition and a thorough diagnosis are the most important factors for successful treatment [2].

Various treatment approaches have been proposed that depend on the age of the patient, the developmental stage of the root, the position of the tooth, the severity of infraocclusion, the number of the teeth affected, the severity of tilting of adjacent teeth, and the presence and location of the permanent successor [3].

It is often difficult to decide exactly the starting point of treatment. In the case of a cooperative patient with regular recall periods, a watchful waiting approach maybe the best. Moreover, if there is no evidence of unusual caries problems or loss of arch length, the dentist may choose to keep the tooth under observation [2].

A conservative treatment approach for ankylosed primary teeth is the continuous supervision of tooth eruption evidenced with periodic radiographic observation of normal root resorption [9, 10].

A tooth that is definitely ankylosed may surprisingly undergo root resorption at a given time and be normally shed. In this case, it may seem logical not to extract all the infraoccluded teeth but instead to follow rigorously, as there is a chance for it in the eruption path. However, in this patient, because of severe infraocclusion of the second primary molar and the risk of space loss in the near future while the first permanent molar erupts, immediate intervention was considered.

Another conservative treatment option was composite buildup or a stainless steel crown to prevent tipping of adjacent teeth and to restore the occlusion to the correct height, thereby preventing the tooth of the opposing arch from supraeruption. However, for this case, because the infraocclusion was severe, it was imprudent and even impossible to buildup the occlusal table with either composite resin or a stainless steel crown.

Extraction as an eventual treatment, preferably as early as possible [4, 5], is recommended by many authors. Other authors recommend this treatment only in the case of severely affected primary molars and where the evidence of possible future crowding is observed [11]. Early extraction is only advisable when there is occlusal disturbance with severe tipping of adjacent teeth and malposition of the permanent succedaneous combined with severe infraocclusion [7]. However, early extraction may be technically difficult and may result in root fractures or disturbance of the successor tooth bud [3, 12]. In this case, due to the five-year-old patient's fear of surgery and the parents' insistence on adopting a more conservative treatment as well as the possible complications of surgical extraction such as bone loss, this method was not considered, and so luxation was considered.

Luxation is a treatment option for ankylosed teeth, permitting the teeth to continue its eruption. The theory behind luxation of affected primary molars is that the bony union between the alveolus and the ankylosed teeth can be broken [3].

Biederman as well as Skolnick have reported the efficacy of a luxation technique in breaking bony ankylosis [13, 14]. If this technique is not immediately successful, then it can be repeated in six months [2].

Lygidakis et al. adopted surgical luxation and elevation as a treatment approach for a localized secondary eruption failure of the mandibular right first permanent molar [15]. This treatment included surgical luxation of the tooth, followed by elevation to the occlusal plane and stabilization to the adjacent teeth. A three-year followup showed a successful result without considering the clinical or radiological pathology of the area [15].

Although luxation to free ankylosed permanent teeth (not generally recommended) and the absence of a case report introducing luxation as a treatment approach for ankylosed primary molars have been attempted with limited success [8], in this present case, we decided to luxate the ankylosed tooth. 

Despite the limited success of the luxation method in ankylosed permanent teeth [8], in this patient, the submerged primary molar reached the occlusal plane after luxation by elevator after four months.

## 4. Conclusion

Although the majority of ankylosed teeth with permanent successors shed normally, early and proper intervention to prevent occlusal discrepancies is advisable. There are several management methods in case of ankylosed primary molars, and from these methods, luxation can be considered a safe and effective treatment approach for the management of infraoccluded primary molars.
